# Mechanical Properties of Single-Crystal Calcite and Their Temperature and Strain-Rate Effects

**DOI:** 10.3390/ma15134613

**Published:** 2022-06-30

**Authors:** Chaocai Luo, Xinhua Yang, Jie Li

**Affiliations:** 1 School of Aerospace Engineering, Huazhong University of Science and Technology, Wuhan 430074, China; luochaoc@hust.edu.cn (C.L.); jieli_mech@hust.edu.cn (J.L.); 2 School of Transportation, Civil Engineering and Architecture, Foshan University, Foshan 528200, China

**Keywords:** calcite, mechanical properties, deformation mechanism, molecular dynamics

## Abstract

Calcite is the most stable crystalline phase of calcium carbonate. It is applied or found in composite products, the food industry, biomineralization, archaeology, and geology, and its mechanical properties have attracted more and more attention. In this paper, the mechanical behaviors of single-crystal calcite under uniaxial tension in different directions were simulated with the molecular dynamics method. The obtained elastic moduli are in good agreement with the experimental results. It has been found from further research that single-crystal calcite has typical quasi-brittle failure characteristics, and its elastic modulus, fracture strength, and fracture strain are all strongly anisotropic. The tensile failure is caused by dislocation emission, void formation, and phase transition along the [010] and [421] directions, but by continuous dislocation glide and multiplication along the $\left[42\bar{1}\right]$ direction. The fracture strength, fracture strain, and elastic modulus are all sensitive to temperature, but only elastic modulus is not sensitive to strain rate. The effects of temperature and logarithmic strain rate on fracture strength are in good agreement with the predictions of fracture dynamics.

## 1. Introduction

Calcium carbonate (CaCO_3_) is one of the most common of the mineral materials that provide important support for human life and industry. It occurs naturally in a wide variety of organisms, such as coccolithophores, foraminifers, and corals, as well as pearl oysters and animal bones [1,2]. Due to its biocompatibility and ease of use, calcium carbonate plays a very important role in biomedical aspects [3,4,5], such as regeneration [6,7]. It is also a biological mineral. When copolymer is added, its friction properties and other mechanical properties can be greatly improved [8,9]. In addition, it plays a key role in many anthropological and geological studies [10], materials protection [11], and accurate dating [12,13,14,15].

Calcium carbonate has three anhydrous crystal types, including calcite, aragonite, and vaterite. As a building material, calcite is found throughout nature. According to the statistics from European Calcium Carbonate Association (CCA–Europe), the global construction industry consumes hundreds of millions of tons of calcium carbonate every year. In addition, calcite accounts for more than 60% of the content of polymer fillers and is used to reinforce raw materials [16]. The addition of calcite not only improves the characteristics of raw materials, but also greatly reduces the product cost. These applications are inevitably affected by temperature and strain changes. Therefore, it is of great significance to understand the mechanical properties of calcite at different temperatures and strains.

At present, the research on calcite, from Bridgeman’s pioneering work [17] to many static and dynamic experimental studies [18,19,20,21,22,23,24], and then to the recent atomic scale simulations of phase-transition processes [25,26,27,28], mainly focuses on its high-pressure phase-transition behavior. In addition, Sokolá et al. [29] studied the effects of calcite content on the mechanical properties of calcium carbonate at different temperatures. They found that calcite can reduce the shrinkage of samples during sintering, which can improve the characteristics of samples. Kunitake et al. [30] obtained biological calcite from the prismatic layer of mollusks and compared the hardness and modulus of biological calcite and geological calcite through an indentation experiment. They found that biological calcite has higher hardness, and pointed out that the hardening mechanism in which the dislocation motion is hindered causes the failure of calcite. Subsequently, Schuster et al. [31] twisted the calcite aggregates under high pressure and characterized the products by X-ray diffraction. They found that the dominant slip plane $\left(10\bar{1}4\right)$ has obvious dislocation characteristics.

Although some mechanical properties of calcite have been tested and some deformation mechanisms have been revealed, the research and understanding of these mechanisms are not complete. In this study, we used the molecular dynamics (MD) method to simulate the mechanical behavior of calcite under uniaxial tensile loading, studied the effects of temperature and strain rate on the mechanical properties of calcite, and tried to reveal its internal deformation mechanism at the atomic scale.

## 2. Model and Method

### 2.1. Interatomic Interaction Potential

An interatomic potential function describes the interaction between atoms. In MD simulations, the potential function plays a decisive role in the simulation results. For ionic materials, there are generally two interatomic potential function models: the rigid ion model (RIM) and the shell model (SM). In the RIM, the potential energy only depends on the distance of each atomic pair. In order to more accurately describe the polarization mechanism of negative ions, especially $O^{2-}$, the SM divides each negative ion into two particles, namely a core and a shell [32]. The polarization-coordinate-coupling method is used to shorten the simulation time and improve the convergence speed [33].

For calcite, a lot of RIMs [34,35,36,37,38,39,40] and SMs [35,37,41,42,43] have been developed. They have different descriptions of C–O interaction. Although the SMs show the volume characteristics [41,42] of calcite crystals through the polarization of CO_3_^2−^, the addition of core and shell particles in $O^{2-}$ significantly reduces the efficiency of calculation. Therefore, an available simulation model has a relatively small size.

The SM developed for calcite by Dove et al. [34] can give more consistent predictions of the mechanical properties with the experimental results. In this model, the total potential energy includes bonded and non-bonded parts, namely:

(1) $$V=V_{\mathrm{bonded}}+V_{\mathrm{non}-\mathrm{bonded}}$$

In the bonded part, the intermolecular harmonic function is used to deal with the bond-bending between O–C and C–O in O–C–O, and the out-of-plane potential of the CO_3_^2−^ group is realized by a four-body torsional term. Moreover:

(2) $$V_{\mathrm{bonded}}=\sum_{ijk}\frac{1}{2}k_{\theta ,ijk}{\left(\theta_{ijk}-\theta_{0,ijk}\right)}^{2}+\sum_{ijkl}k_{t,ijkl}[1-\cos(2\varphi_{ijkl})]$$

where $k_{\theta }$ is the bond-bending constant, $\theta_{0}$ is the equilibrium bond angle between O–C and C–O in the O–C–O, taken as 120° here, θ is the actual bond angle, $k_{t}$ is the torsional constant, φ is the angle between two O–C–O planes in CO_3_^2−^ group, taken as 180° here, and subscripts i, j, k, and l represent different atoms.

In the non-bonded part, Born–Mayer interaction potential is used to characterize the short-range interactions of Ca–O, C–O, and O–O, and the Coulomb potential of their long-range interactions. See:

(3) $$V_{\mathrm{non}-\mathrm{bonded}}=\sum_{ij}A_{ij}\exp(-r_{ij}/\rho_{ij})+\frac{1}{4\pi \varepsilon_{0}}\sum_{ij}\frac{q_{i}q_{j}}{r_{ij}}$$

where A and ρ are Born–Mayer potential parameters, $\varepsilon_{0}$ is the dielectric constant, q is the charge of atom, and r is the distance between two atoms with cut-off radius of 10 Å. All the potential function parameters are listed in Table 1.

### 2.2. The Computer Model

**Figure 1:**
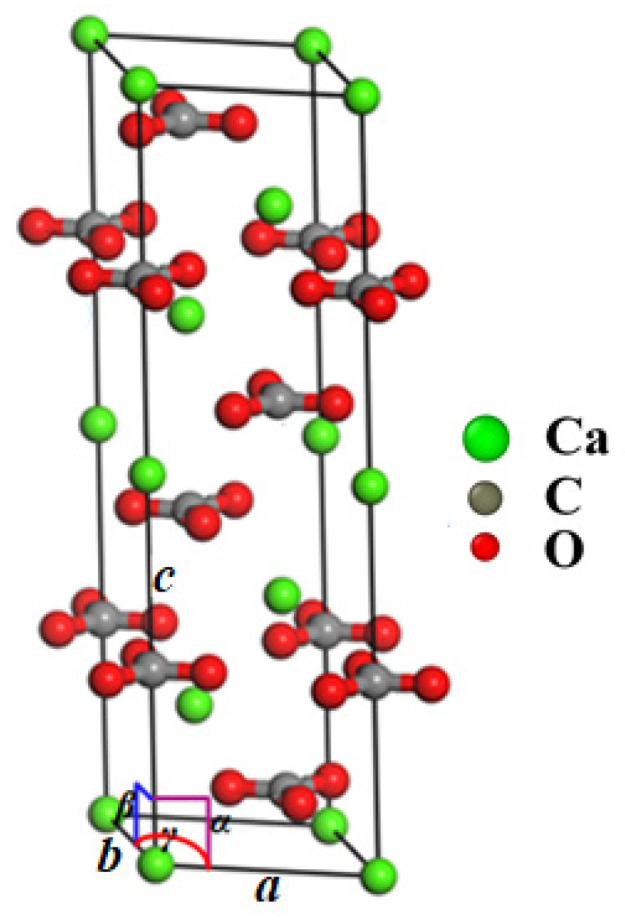
Calcite unit cell (green for Ca, gray for C, and red for O).

**Figure 2:**
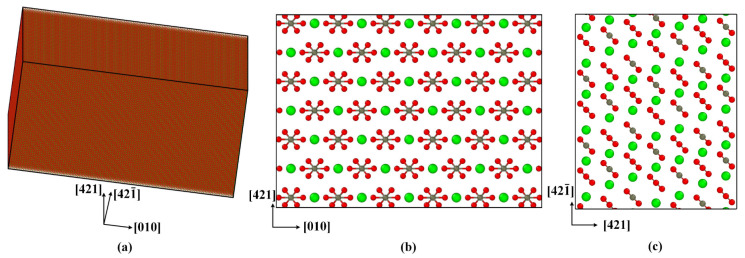
Computer model of single-crystal calcite: (**a**) 3D view, (**b**) top view, and (**c**) side view.

Calcite belongs to the trigonal system, and the space group is $R\bar{3}c$. In a hexagonal calcite unit cell (*a* = *b* = 4.990 Å, *c* = 17.061 Å, *α* = *β* = 90°, and *γ* = 120°), there are 64 atoms (Figure 1). A computer model of single-crystal calcite with a size of 50 nm × 50 nm × 25 nm and 4.8 million atoms in total was constructed by using the unit cell structure proposed by Graf [44], as shown in Figure 2. A Cartesian coordinate system had the [010]*,* [421], and $\left[42\bar{1}\right]$ directions, respectively.

The calculation was performed using the general MD simulation package LAMMPS [45]. The periodic boundary conditions were used along the [010], [421] and $\left[42\bar{1}\right]$ directions. In the NPT ensemble with a constant number of atoms, pressure, and temperature, the model was relaxed for 100 ps to equilibrium at a time step of 1 fs and temperature of 500 K. Then, the model was stretched along the [010], [421] and $\left[42\bar{1}\right]$ directions, respectively. The NPT ensemble was also used in the tension process simulations. The pressure was controlled by air pressure and the temperature by a Nose–Hoover thermostat. The particle–particle–particle–mesh (PPPM) algorithm [46], with accuracy of 10^−6^, was adopted to solve the dynamic equations.

## 3. Results and Discussion

### 3.1. Uniaxial Tensile Behaviors in Different Directions

**Figure 3:**
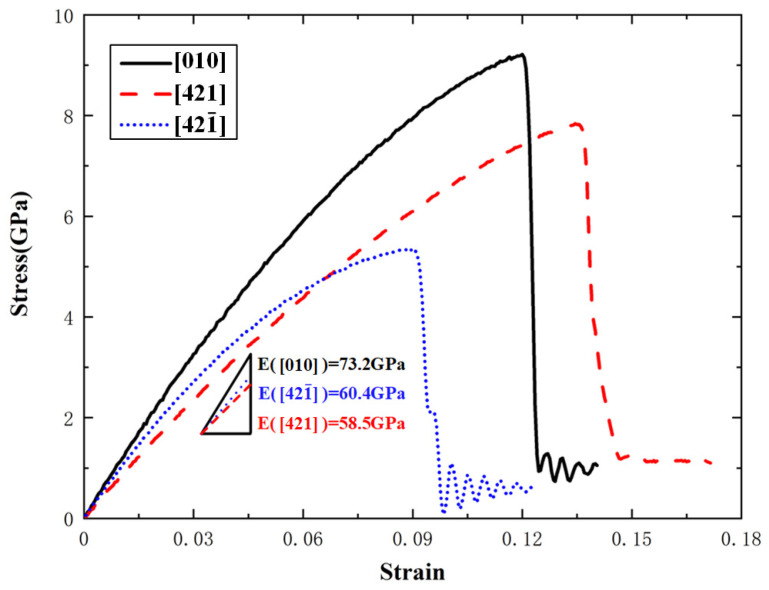
Uniaxial tensile stress–strain curves of single-crystal calcite along the [010], [421], and $\left[42\bar{1}\right]$ directions at 300 K.

Figure 3 shows the uniaxial tensile stress–strain curves along the [010], [421], and $\left[42\bar{1}\right]$ directions at 300 K temperature and 1 × 10^8^ s^−1^ strain rate. It can be seen that although the three stress–strain curves have very similar shapes, they obviously have different slopes of the elastic stage, different ultimate stresses or fracture strength, and different strains corresponding to the ultimate stresses. This reflects the remarkable anisotropy of single-crystal calcite in the mechanical properties. At the stage of elastic deformation, the stress increases with the strain in an approximately linear trend. When the fracture strength is reached, the curves enter into the fracture stage. The results indicate that the tensile failure behavior of single-crystal calcite exhibits typical quasi-brittleness characteristics along the three directions [47]. By fitting the elastic deformation stage with the least square method, the elastic modulus along the [010] direction is about 73.2 GPa, which is very close to the experimental result of 70.23 ± 1.35 GPa [48]. Moreover, the elastic moduli along the [421] and $\left[42\bar{1}\right]$ directions are 58.5 GPa and 60.4 GPa respectively. They are about 20% smaller than that along the [010] direction.

**Figure 4:**
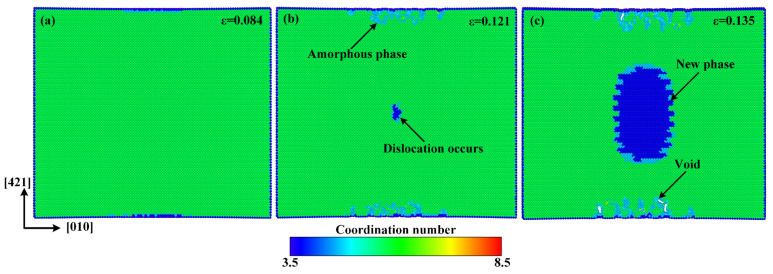
Atomic configurations when the tensile strain along the [010] direction reaches: (**a**) *ε* = 0.084, (**b**) *ε* = 0.121, and (**c**) *ε* = 0.135. The atoms are colored according to the coordination number of Ca with green for calcite, blue for the new phase, and cyan for the amorphous phase.

**Figure 5:**
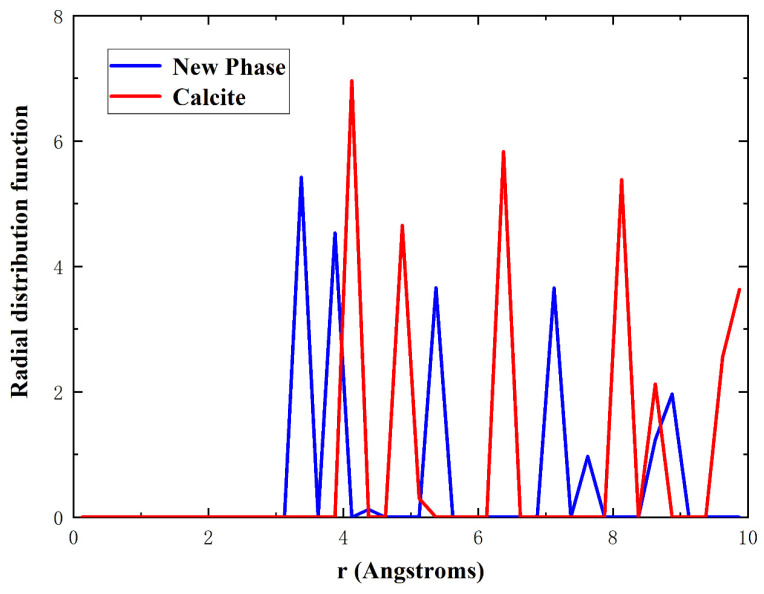
Radial distribution functions of Ca–Ca for calcite in red and the new phase in blue.

In order to understand the atomistic mechanism of tensile deformation and failure behavior of single-crystal calcite, we extracted the atomic configurations at different stages of tensile deformation along the [010] direction, as shown in Figure 4. In this study, the radial distribution function (RDF) as well as the atomic coordination number was used to identify a new phase, as shown in Figure 5. The RDF analysis shows that Ca–Ca has the nearest-neighbor distance of 3.2 Å in the new phase, while it is 3.8 Å in calcite, which corresponds to the first peaks of RDFs in Figure 5. As an intermediate value between 3.2 Å and 3.8 Å, therefore, 3.5 Å was chosen as the cut-off radius to calculate the coordination number of calcium atoms. Figure 4a–c shows the atomic configurations when the strain reaches 0.084 in the elastic deformation stage, 0.121 at the rapid stress reduction stage, and 0.135 at the failure stage, respectively. It can be seen that at the elastic deformation stage, only some dislocations initiate from the middle of both sides along the [421] direction. After the curve enters into a sharp stress-declining stage with the increase of strain, the dislocations in both the sides develop inwards and an amorphous phase forms gradually. Meanwhile, dislocations are also initiating, as shown in Figure 4b. At the failure stage, the dislocations develop rapidly along the slip plane $\left(10\bar{1}4\right)$. As the most important cleavage plane of calcite crystals [49,50,51], it is orthogonal to the [421] direction. As the strain increases, the further development of dislocation leads to the occurrence of voids in the middle of both sides along the [421] direction, while an ordered new phase is constantly formed in the center of the model, as shown in Figure 4c. Although the new phase is different from calcite, it has a regular atomic arrangement and a fixed radial distribution function, as shown in Figure 5. For observation, the atoms are colored according to the coordination number of calcium atoms in Figure 4, with green for calcite, blue for the new phase, and cyan for the amorphous phase, respectively. The development of the new phase and voids is the fundamental reason for the rapid stress reduction at the second stage [47]. The failure process of calcite in tension along the [010] direction originates from initial dislocation emission and ends with the rapid development of the new phase and voids.

**Figure 6:**
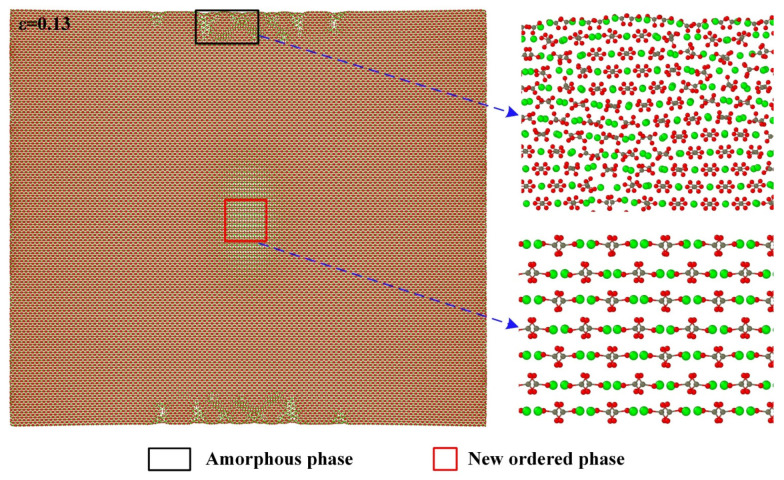
Partial enlarged details of atomic configuration at strain of 0.13 along the [010] direction.

Figure 6 shows the atomic configuration when the strain reaches 0.13 and its local enlarged diagrams for the atomic arrangements in the middle of both sides along the [421] direction and the center of the model. It can be seen that, similar to the case for the [010] direction, the amorphous phase also develops in the middle of both sides along the [421] direction and the new ordered phase forms in the center of the model.

**Figure 7:**
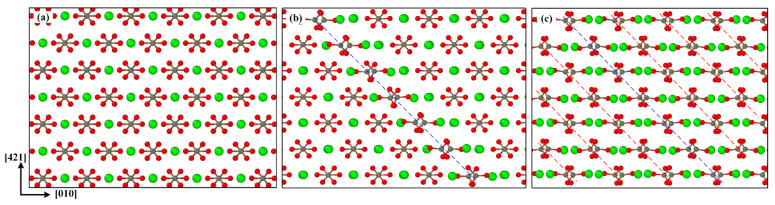
Dislocation initiation and development: (**a**) initial perfect calcite structure, (**b**) a dislocation line formed at the rapid stress reduction stage, (**c**) a new ordered phase formed by dislocation development.

In order to understand the internal mechanism of dislocation slip induced by the phase transition in uniaxially stretched calcite, Figure 7 shows the process from the dislocation initiation and development to the formation of the new ordered phase. Compared with the initial perfect calcite structure shown in Figure 7a, when the stress reaches the fracture strength, a dislocation line is formed in the center of the model, as shown in Figure 7b, which corresponds to Figure 4b. In Figure 7c, the rapid propagation and development of dislocations causes the new phase to form. The Ca^2+^ and CO_3_^2−^ interaction inside calcium carbonate molecule is much stronger than the interaction between calcium carbonate molecules, so dislocations easily occur between two adjacent calcium carbonate molecules. As reported in reference [52], they tend to break weaker bonds in the cleavage plane.

**Figure 8:**
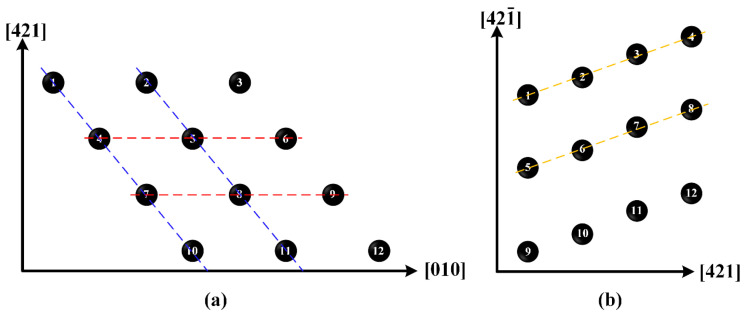
Arrangement of calcium carbonate molecules on (**a**) top plane and (**b**) side plane.

Figure 8 shows the layouts of computer model on the top plane and side plane. In this figure, each point has the average coordinates of the five atoms in the corresponding calcium carbonate molecule. When the model is stretched along the [010] direction, as shown in Figure 8a, the calcium carbonate molecules in the dotted blue lines, such as molecules 1, 4, 7, and 10 or 2, 5, 8, and 11, act as a close-packed plane. There is a high molecular density on these planes. Due to large distance between two neighboring close-packed planes, there is a weak atomic binding force and a small slip resistance between them, so that the slip preferentially occurs.

**Figure 9:**
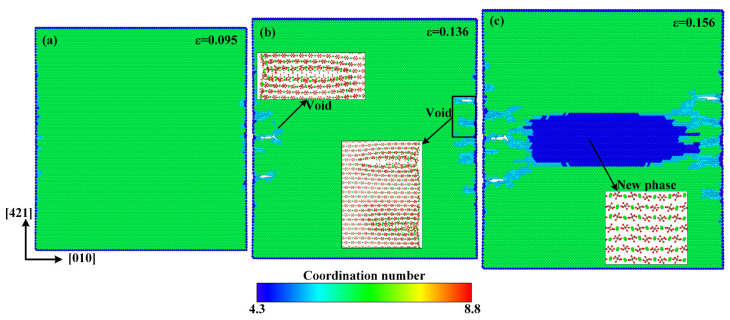
Atomic configurations when the tensile strain along the [421] direction reaches: (**a**) *ε* = 0.095, (**b**) *ε* = 0.136, and (**c**) *ε* = 0.156.

**Figure 10:**
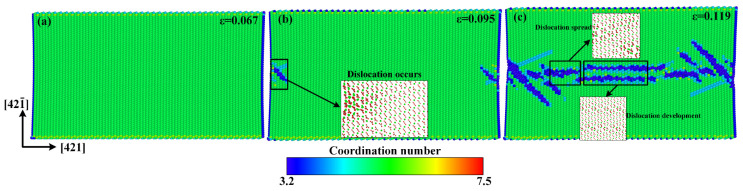
Atomic configurations when the tensile strain along the $\left[42\bar{1}\right]$ direction reaches: (**a**) *ε* = 0.067, (**b**) *ε* = 0.095, and (**c**) *ε* = 0.119.

In order to explain the anisotropic mechanical properties of single-crystal calcite, the atomic configurations of tensile deformation and failure processes along the [421] and $\left[42\bar{1}\right]$ directions are given in Figure 9 and Figure 10, respectively. No defects occur at the elastic deformation stage along the [421] and $\left[42\bar{1}\right]$ directions. Unlike the tensile failure case along the [010] direction, there are a lot of voids that form in advance at the rapid stress reduction stage, as shown in Figure 9b. This phenomenon can also be explained by the arrangement of calcium carbonate molecules. As shown in Figure 8a, the calcium carbonate molecules in the dotted red lines, such as molecules 4, 5, and 6 or 7, 8, and 9, act as a close-packed plane, and are stretched in a direction just perpendicular to the close-packed plane, resulting in a direct separation between the two close-packed planes and the formation of voids. It is more similar to the tensile failure of single-crystal aragonite along the [010] direction [53]. As the strain increases, the voids continue to grow up and phase transition takes place in the center of the model, as shown in Figure 9c. In the new phase, Ca–Ca has the nearest neighbor distance of 3.4 Å, which is different from the phase with the Ca–Ca nearest neighbor distance of 3.2 Å shown in Figure 4c.

A similar quasi-brittle fracture can also be observed when the model is stretched along the $\left[42\bar{1}\right]$ direction. Compared with the atomic configuration at the elastic deformation stage shown in Figure 10a, some dislocations occur on both sides along the [421] direction at the rapid stress-reduction stage, as shown in Figure 10b, and then rapidly multiply and are emitted to the center of the model at the failure stage, as shown in Figure 10c. As shown in Figure 8b, the calcium carbonate molecules in the dotted yellow lines, such as molecules 1, 2, 3, and 4; or 5, 6, 7, and 8, act as a close-packed plane. Different from the tensile failure cases along the [010] and [421] directions, there is no new phase to initiate in the whole tensile failure process along the $\left[42\bar{1}\right]$ direction.

The anisotropy of single-crystal calcite is not only reflected in the stress–strain curves, but also in the deformation and failure processes. When the model is stretched along the [010] and [421] directions, the rapid stress reduction is caused by dislocation initiation and subsequent phase transition and void formation. When the model is stretched along the $\left[42\bar{1}\right]$ direction, however, the stress drop is caused by dislocation initiation in both the sides parallel to the $\left[42\bar{1}\right]$ direction and then emission to the center of the model.

### 3.2. Influence of Temperature and Strain Rate

**Figure 11:**
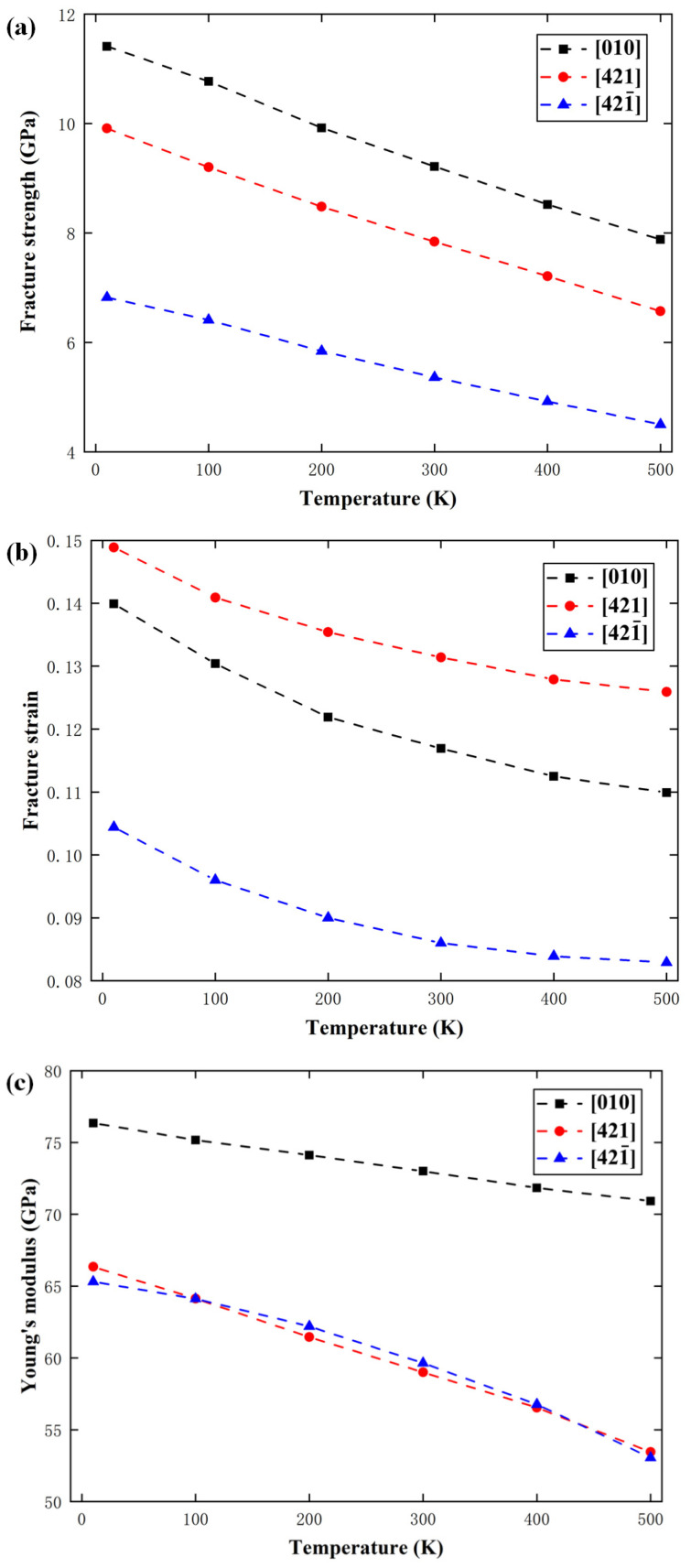
Variations of (**a**) fracture strength, (**b**) fracture strain, and (**c**) elastic modulus with temperature.

As with other inorganic materials, the mechanical properties of calcite are affected by temperature and strain rate [54,55]. In order to study the effect of temperature on the deformation behavior, the strain rate was fixed at 1 × 10^8^ s^−1^ but the temperature was changed from 10 K to 100 K, 200 K, 300 K, 400 K, and 500 K. Figure 11 shows the variations of fracture strength, fracture strain, and elastic modulus with temperature along the [010], [421], and $\left[42\bar{1}\right]$ directions. At 10 K, the fracture strength, fracture strain, and elastic modulus are 11.41 GPa, 0.140, and 76.35 GPa along the [010] direction, respectively; they are 9.91 GPa, 0.149, and 66.34 GPa along the [421] direction, respectively; but they are 6.82 GPa, 0.104, and 65.31 GPa along the $\left[42\bar{1}\right]$ direction, respectively. Obviously, the mechanical properties are significantly different along different directions, so they are strongly anisotropic. With elevated temperature, the fracture strength, fracture strain, and elastic modulus are all reduced. When the temperature is elevated from 10 K to 500 K, the fracture strength reduces by 32.3%, 34.1%, and 33.8% along the [010], [421], and $\left[42\bar{1}\right]$ directions, respectively, the fracture strain reduces by 23.3%, 22.6%, and 21.1%, respectively, and the elastic modulus reduces by 12.6%, 19.5%, and 19.1%, respectively.

**Figure 12:**
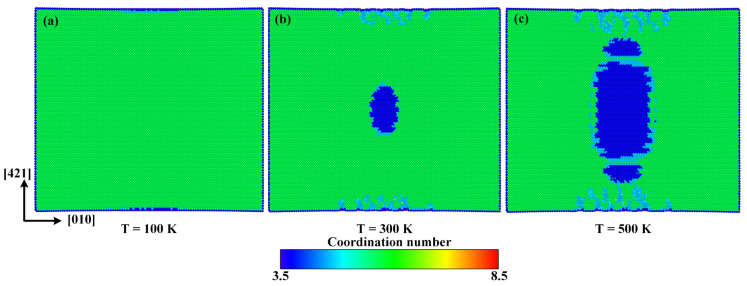
Atomic configurations when the strain reaches 0.125 at (**a**) 100 K, (**b**) 300 K, and (**c**) 500 K, respectively.

In order to investigate the internal mechanisms of temperature effect, the atomic configurations at 100 K, 300 K, and 500 K when the tensile strain reaches 0.125 along the [010] direction are given in Figure 12. It can be seen that when the temperature elevates from 100 K to 300 K and 500 K, the volume of the new phase expands rapidly, which should be the main reason for the decline of fracture strength, fracture strain, and elastic modulus caused by the elevated temperature.

**Figure 13:**
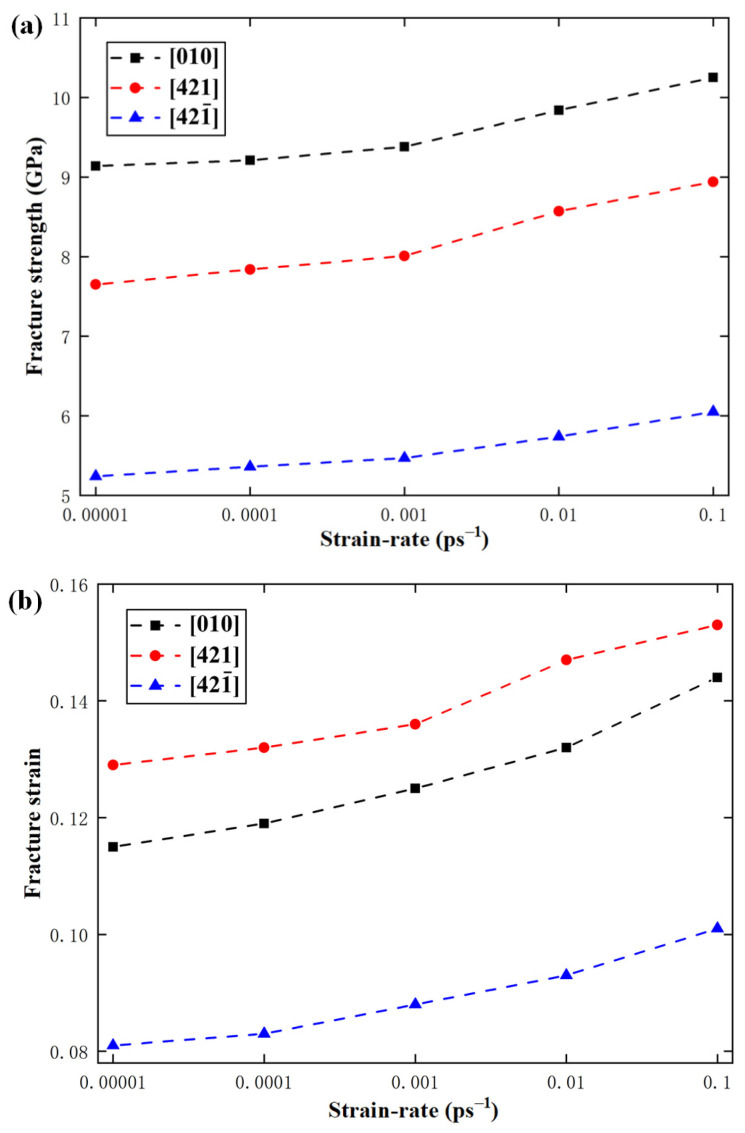

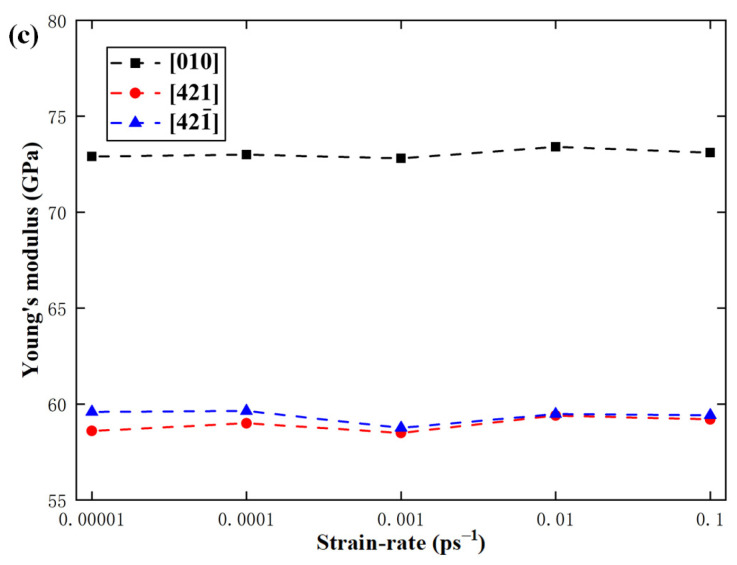
Variations of (**a**) fracture strength, (**b**) fracture strain, and (**c**) elastic modulus with strain rate.

In order to study the effect of strain rate on the mechanical properties of calcite, the temperature was fixed at 300 K, but the strain rate was changed from 0.00001 ps^−1^ to 0.0001 ps^−1^, 0.001 ps^−1^, 0.01 ps^−1^, and 0.1 ps^−1^. Figure 13 shows the variations of fracture strength, fracture strain, and elastic modulus with the strain rate along the [010], [421], and $\left[42\bar{1}\right]$ directions. It can be seen that with the increase of strain rate from 0.00001 ps^−1^ to 0.1 ps^−1^, the fracture strength along [010], [421], and $\left[42\bar{1}\right]$ directions increases by 10.8%, 14.4%, and 13.4% respectively, and the fracture strain along the directions increases by 16.6%, 15.6%, and 17.2% respectively. It can be seen that both the fracture strength and fracture strain increase monotonously with the increase of strain rate. However, the strain rate hardly has any effect on the elastic modulus.

**Figure 14:**
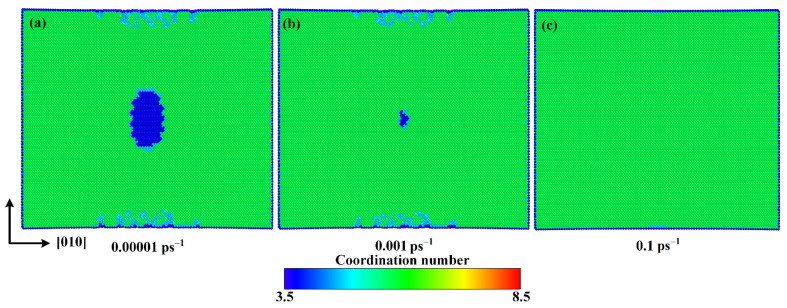
Atomic configurations at 0.125 strain for strain rates of (**a**) 0.00001 ps^−1^, (**b**) 0.001 ps^−1^, and (**c**) 0.1 ps^−1^.

In order to investigate the internal mechanisms of strain rate effect, the atomic configurations with tensile strain of 0.125 along the [010] direction are given in Figure 14 for strain rates of 0.00001 ps^−1^, 0.001 ps^−1^, and 0.1 ps^−1^. It can be seen that with the increase of strain rate, the volume of the new phase is noticeably reduced, which should be the main reason for the increase of the fracture strength and fracture strain caused by increasing strain rate. In general, the increase of strain rate leads to a shorter time for the atoms in calcite to respond to the load, so that they have less chance to overcome the energy barrier and destroy chemical bonds.

### 3.3. Fracture Dynamics Analysis

Fracture dynamics can explain the temperature and strain rate effects of bond failure through thermal activation. Arrhenius regarded the life cycle τ as the following function of tensile stress σ and temperature T [56]:

(4) $$\tau (\sigma ,\;T)=\tau_{0}\exp\left(\frac{U_{0}-c\sigma V}{kT}\right)$$

where $\tau_{0}$ is the average vibration period of atoms, $k=1.381\times 10^{-23}\;J/K$ is the Boltzmann constant, $U_{0}$ is the dissociation energy of interatomic chemical bonds, V is the activation volume, and c is the local overstress coefficient.

In a uniaxial tensile process, the stress changes with time, that is σ=σ(t). According to Bailey’s principle [57], the fracture time $t_{r}$ can be obtained by:

(5) $$\int_{0}^{t_{r}}\frac{dt}{\tau [\sigma \left(t\right),\;T]}=1$$

For linear elastic materials, we have:

(6) $$\sigma \left(t\right)=E\varepsilon \left(t\right)=E\dot{\varepsilon }t$$

(7) $$\sigma_{f}=E\dot{\varepsilon }t_{r}$$

By substituting Equations (4), (6), and (7) into Equation (5), the fracture strength can be given by:

(8) $$\sigma_{f}=\frac{kT}{\gamma }\ln\dot{\varepsilon }+\left[\frac{U_{0}}{\gamma }-\frac{kT}{\gamma }\ln\left(\frac{kT}{\gamma \tau_{0}E}\right)\right]$$

where γ=cV. This shows that there is a linear relationship between the fracture strength and the logarithmic strain rate when the temperature is fixed.

Equation (8) can be rewritten as:

(9) $$\sigma_{f}=-\frac{k}{\gamma }\ln{\left(\frac{\gamma E\dot{\varepsilon }\tau_{0}}{kT}\right)}^{-1}T+\frac{U_{0}}{\gamma }$$

If $\dot{\sigma }≐10^{7}\;\mathrm{Pa}/s$ and $\tau_{0}≐10^{-13}s$ [58], which is close to the average vibration period of solid atoms, $\ln{\left(\gamma E\dot{\varepsilon }\tau_{0}/kT\right)}^{-1}$ would have a value between 26.02 and 26.71 when the temperature is changed from 10 K to 500 K. Therefore, $\ln{\left(\gamma E\dot{\varepsilon }\tau_{0}/kT\right)}^{-1}$ is nearly temperature-independent in this temperature range [59]. Let $\ln{\left(\gamma E\dot{\varepsilon }\tau_{0}/kT\right)}^{-1}=26.3$, thus Equation (9) can be reduced to:

(10) $$\sigma_{f}=-26.3\frac{k}{\gamma }T+\frac{U_{0}}{\gamma }$$

**Figure 15:**
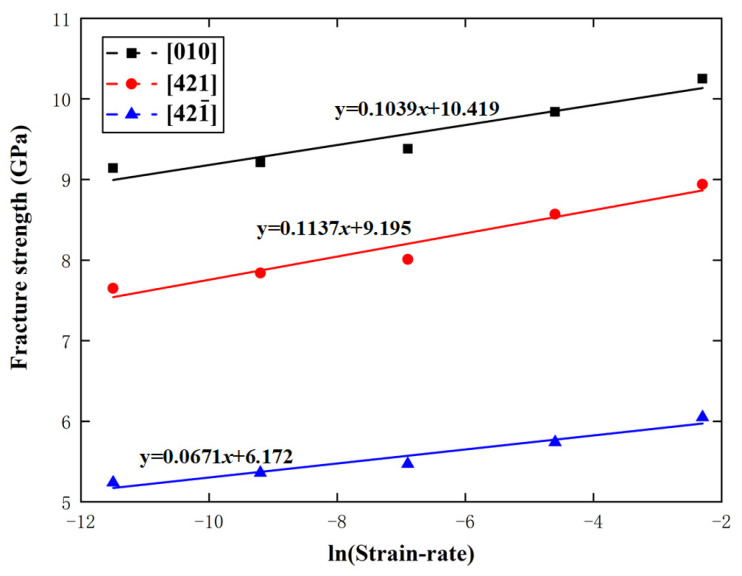
Variations of fracture strength with logarithmic strain rate along the [010], [421], and $\left[42\bar{1}\right]$ directions.

**Figure 16:**
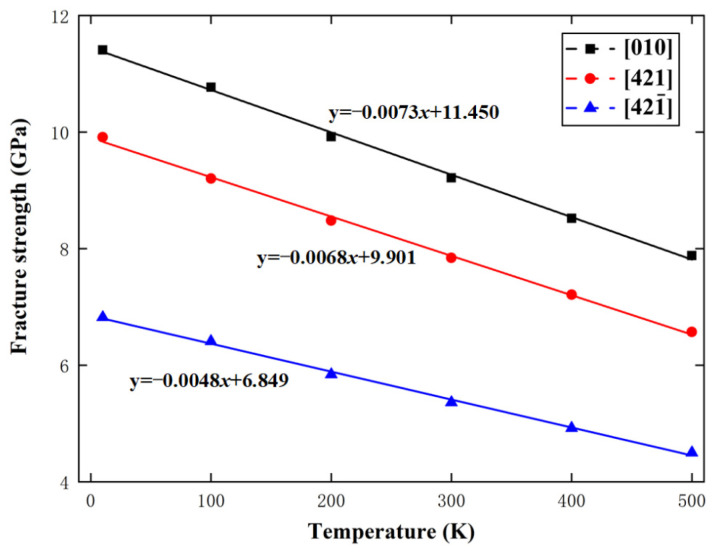
Variations of fracture strength with temperature along the [010], [421], and $\left[42\bar{1}\right]$ directions.

For calcium carbonate, $\gamma =5.67\times 10^{-29}m^{3}$. When T=300 K, $\frac{kT}{\gamma }=0.073\;\mathrm{GPa}$ in Equation (8), which lies in the range of 0.0671 to 0.1137 GPa, given by the above MD simulations. In addition, $-26.3\frac{k}{\gamma }=-0.0064\;\mathrm{GPa}$ in Equation (10), which is between −0.0073 GPa and −0.0048 GPa, given by the above MD simulations. Figure 15 and Figure 16 show the variations of calcite fracture strength with logarithmic strain rate and temperature along the [010], [421], and $\left[42\bar{1}\right]$ directions, respectively, with the predictions from Equations (8) and (10). Obviously, the MD simulations are in good agreement with the fracture dynamics analysis.

## 4. Conclusions

In this paper, the mechanical properties of single-crystal calcite, as well as their temperature and strain-rate effects, were studied by MD simulations. Single-crystal calcite’s tensile failure behavior exhibits typical quasi-brittleness characteristics. The elastic moduli obtained by the simulations are very close to the experimental value, which confirms the reliability of MD simulations. The fracture strength, fracture strain, and elastic modulus are all highly anisotropic. The fracture strength and elastic modulus along the [010] direction is much higher than those along the [421] and $\left[42\bar{1}\right]$ directions. High anisotropy is caused by different failure and deformation mechanisms in different directions, including dislocation initiation and development, phase transition, and void formation. It is found that the fracture strength, fracture strain, and elastic modulus are all very sensitive to temperature, but only elastic modulus is not sensitive to strain rate. The variations of fracture strength with temperature and logarithmic strain rate are in good agreement with the predictions of fracture dynamics.

## Figures and Tables

**Table 1:** Potential function parameters [34].

Charges (|e|)		
Ion	*q*	
Ca	+1.64203	
C	+1.04085	
O	−0.894293	
Short-range potentials		
Interaction	A (eV)	ρ (Å)
Ca–O	3943.5977	0.251570
O–O	2879.1262	0.252525
C–O	1.7411 × 10^15^	0.03873
Force constants		
Bond-bending constant for O–C–O $k_{\theta }$ (eV rad^−2^)		3.69441
Torsional constant $k_{t}\;(\mathrm{eV})$		0.125125

## Data Availability

The original contributions presented in the study are included in the article; further inquiries can be directed to the corresponding author.
